# ‘It opened my eyes, my ears, and my heart’: Codesigning a substance use disorder treatment programme

**DOI:** 10.1111/hex.13908

**Published:** 2023-11-03

**Authors:** Julie Bosak, Mari‐Lynn Drainoni, Cheri Bryer, Daisy Goodman, Lisa Messersmith, Eugene Declercq

**Affiliations:** ^1^ Community Health Services Boston University School of Public Health Boston Massachusetts USA; ^2^ Dartmouth Hitchcock Medical Center Lebanon New Hampshire USA; ^3^ Dartmouth Geisel School of Medicine Lebanon New Hampshire USA; ^4^ Department of Medicine, Section of Infectious Diseases Boston University Aram V. Chobanian & Edward Avedisian School of Medicine Boston Massachusetts USA; ^5^ Department of Health Law Policy and Management Boston University School of Public Health Boston Massachusetts USA; ^6^ Department of Global Health Boston University School of Public Health Boston Massachusetts USA

**Keywords:** addiction treatment service design, codesign, experience based codesign, i‐PARIHS, pregnant and parenting women in recovery

## Abstract

**Background:**

Pregnant and parenting women have low engagement and poor retention in substance use disorder (SUD) treatment. The aim of this study was to analyse the implementation of an adapted experience‐based codesign (EBCD) process involving SUD treatment staff and pregnant or parenting women with lived experience (WWLE) of SUD to launch a residential treatment service where women could coreside with their children and receive long term comprehensive treatment for dual diagnosis of SUD and mental illness.

**Methods:**

A process evaluation was conducted utilising five data sources: two sets of semistructured interviews with WWLE and SUD treatment staff, ethnographic observation and transcripts from group events, and meeting minutes. Based on the Integrated Promoting Action on Research in Health Services framework constructs (context, recipients, facilitation, innovation) researchers applied thematic analysis to determine main themes within each construct.

**Results:**

The full sample across the implementation totalled 34 individuals (WWLE = 13 and SUD staff = 21). The EBCD process engaged both cohorts and supported group cohesion and collaborative brainstorming. WWLE felt respected, emotionally safe to share, and empowered by participation. A cohesive, multidisciplinary codesign planning group, inclusive of WWLE, supported a more equitable codesign process. The need for a virtual platform due to the COVID‐19 pandemic impeded human connection and relationship building. The complex environment of residential regulations and uncertainties during start‐up phase of an organisation presented implementation challenges.

**Conclusion:**

These results highlight the feasibility of, and challenges to, effectively engaging WWLE in a codesign process. The findings also demonstrated a positive influence on WWLE's feelings of empowerment. Identified themes reinforce the purposeful components within EBCD that enhance participation, along with new insights to inform successful codesign with a vulnerable population. The author's team included a WWLE who collaborated throughout the full scope of the research process, enriching the overall research and ensuring the authenticity of the presentation of women in recovery's perspective. Utilising the codesign approach to design and implement new services should improve health equity by enhancing patient engagement and retention in care.

**Patient Contribution:**

Parenting WWLE of residential SUD treatment were involved in the full scope of the research process and the implementation being evaluated. For the actual codesign work WWLE were key members of the codesign planning team that met weekly throughout the implementation to plan, implement, problem solve and adapt the process over an 18 month timeframe. As is appropriate for codesign the actual ongoing workgroup participants had average 50% WWLE participation. For the research team, this research is a culmination of the lead author's doctoral dissertation. One member of the five‐person dissertation committee was a recovery coach and a WWLE. She was an active participant across the entire research process overseeing and influencing the research design, conduct of the study, analysis, interpretation of findings and approval of the final manuscript. The findings were member checked with the larger codesign planning group that had additional WWLE members.

## 1 INTRODUCTION

Pregnant and parenting women consistently have low utilisation of and retention in substance use disorder (SUD) treatment.^1^, ^2^ Women with SUD present with significant histories of trauma, with more than a third having a history of intimate partner violence inclusive of physical and sexual trauma.^3^ This history or ongoing experience of trauma, coupled with internalised stigma and shame related to their substance use, can exacerbate a woman's substance use and impede her ability to access, engage in, and sustain appropriate SUD treatment.^4^ While pregnancy may enhance motivation and commitment to treatment, pregnant and parenting women suffer from significantly more barriers, such as fear of child welfare involvement, than the nonparenting population.^5^, ^6^, ^7^ Experts recommend finding innovative approaches to enhance engagement for this population and improve their health equity.^6^, ^8^, ^9^, ^10^, ^11^, ^12^

Codesign is an innovative process that ideally integrates equal input from both care providers and service recipients, who jointly identify issues and then collaboratively design an improved approach to care. The outcome should be a more patient‐centred approach, since codesign proactively incorporates the service users' needs into the design.^13^, ^14^, ^15^, ^16^, ^17^ A well‐executed codesign process integrates trauma‐informed care (TIC) principles, a foundational approach for SUD treatment, including empowerment, voice, choice, collaboration and mutuality.^18^, ^19^ Integrating these principles counteracts the structural power dynamic and encourages meaningful participation, allowing codesign to realise its full benefits. As defined by Lindblom et al.^20^ meaningful participation means each participant must feel safe to share honestly and engage in fully collaborative problem solving.^20^ Codesign presents an opportunity for both positive short‐term outcomes at the individual participant level and long‐term outcomes at the organisational level through improved engagement and retention.^21^, ^22^ For WWLE of SUD collaborating in a well‐executed codesign initiative has the potential to positively influence their recovery through reinforcing multiple TIC principles, enhancing their self‐esteem and self‐efficacy.^23^, ^24^

**Figure 1:**
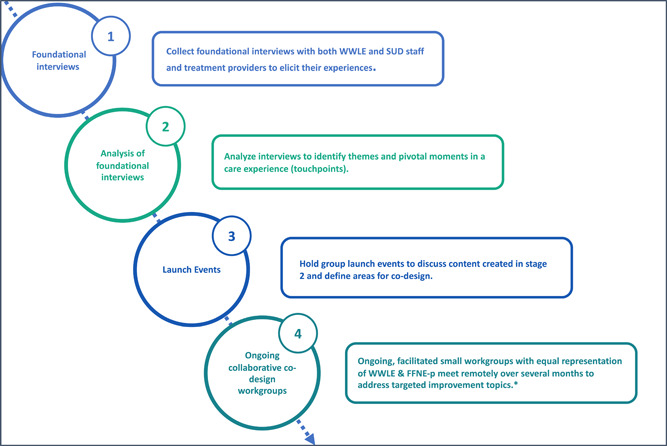
Overview and description of adapted EBCD (see Supporting Information material for more details). *FFNE workgroup topics: visitor and phone policies, work flows for treatment intake, and orientation process. EBCD, experience‐based codesign; FFNE, Families Flourish Northeast.

The primary aim of this study was to evaluate the implementation of an adapted experience‐based codesign (EBCD) process involving SUD treatment staff and pregnant or parenting women with SUD. To our knowledge, codesign with women with SUD and treatment staff has not been previously studied. EBCD is a codesign framework with specific stages (Figure 1).^25^, ^26^ Understanding the feasibility of codesign implementation with this specific vulnerable population fills a research gap and enhances the ability to disseminate an approach that supports their health equity.^16^, ^27^

## 2 METHODS

### 2.1 Study context

This study took place in the context of programme development work for Families Flourish Northeast (FFNE), a nonprofit established in 2019 to create a long‐term residential SUD treatment service for pregnant and parenting women to coreside with their children in northern New England.

### 2.2 The adapted EBCD process

The EBCD process is purposefully crafted to enhance meaningful participation of vulnerable voices typically not part of the decision making.^17^ To fit local context, FFNE compressed the typical eight stages of the EBCD process to four (Figure 1), an adaptation proven effective in previous EBCD research.^25^, ^26^ Using EBCD for programme creation is a new application from the original quality improvement within a specific existing service. Given the uniformity among residential SUD treatment programmes this codesign was approached as a system improvement. Framing the codesign with this lens made it feasible to start the EBCD process with a gathering of individuals' experiences at different programmes and finding common moments that shaped care experiences to focus on for this new programme development.

### 2.3 Participant recruitment

The WWLE were mostly invited from local outpatient SUD treatment programmes and provided a $40/session e‐gift card for their time. Inclusion criteria for the WWLE required previous attendance of a residential treatment programme when they were either pregnant or parenting. The FFNE‐p cohort were either individuals associated with the implementing organisation or other clinical sites outside the state using purposive snowball sampling through the lead researcher's professional networks. Additional inclusion criteria for the participants from outside the state required experience with some version of integrating patient voices into programme management at their residential or outpatient programmes.

### 2.4 Study design and conceptual framework

A process evaluation of the codesign implementation was conducted (Figure 1) guided by the Integrated Promoting Action on Research in Health Services (i‐PARIHS) which incorporates four domains: innovation, facilitation, recipients and context.^28^, ^29^ For this evaluation, the *innovation* was the EBCD process. The *facilitation* construct was considered to be the workgroup facilitators, on‐site champions, and other components necessary to facilitate uptake.^30^ The *recipients* construct encompasses the impact on the process of participating individuals' characteristics, including their motivation, values, beliefs, goals, existing dynamics of power and authority, and knowledge.^29^ The *context* was the implementing organisation and the broader regulatory environment in which it operated.

### 2.5 Data sources

Data included the five data sources detailed in Table 1. Foundational interview guides for both WWLE and providers explored prior SUD treatment experiences for the actual codesign work and insights or concerns for undertaking codesign with this population. Evaluation interviews aimed to elicit the perspectives of codesign workgroup participants regarding the codesign process, power dynamics, respect for different ideas, and aspects that impacted participation.

**Table 1:** EBCD stage matched with data sources, description and collection.

EBCD stage	Data source	Description	Collection
Stage 1	Semistructured foundational interviews (*n* = 32).	Transcripts of interviews with WWLE and SUD treatment staff eliciting their experiences and ideas for improvements.	Lead researcher conducted interviews via Zoom with two different cohorts: WWLE and SUD treatment staff (FFNE‐p). Transcribed by NVivo‐12.
Stage 3	Two launch events ethnographic participant observation.	*Ethnographies* written by lead researcher and research assistant from the video recording of group events.	Launch event video recorded on Zoom.
Stage 3	Two launch events discussion transcripts.	Transcribed conversation from the group events.	Launch event audio recorded on Zoom and transcribed by lead researcher.
Stage 4	Evaluation interviews (*n* = 15).	Transcripts of semistructured interviews with all workgroup participants.	Research assistant completed evaluation interviews with all ongoing workgroup participants and key organisation staff via Zoom. Professionally transcribed.
All stages	Organisation meeting materials.	Power point slides from group meetings and minutes during codesign implementation	Obtained from organisation by lead researcher.
All stages	Lead researcher personal reflective memoing notes.	Notes written following meetings detailing events, personal thoughts and alternative interpretations.	Lead researcher (J. S. B.) wrote *memos* following relevant meetings.

Abbreviations: EBCD, experience‐based codesign; FFNE, Families Flourish Northeast; SUD, substance use disorder; WWLE, women with lived experience.

### 2.6 Research team positionality

This researcher (J. S. B.), a practicing nurse‐midwife with doctoral‐level qualitative research training, had a dual role as lead researcher and organiser of the codesign planning group. For the ethnographies, the lead investigator reviewed the recording from an emic perspective as a participant observer, while the research assistant reviewed the recordings from an etic or outsider's perspective.^31^, ^32^

This research was approved by both Boston University Medical Campus and Dartmouth Hitchcock Medical Centre IRB as exempt Human Subjects research.

### 2.7 Data analysis

The team employed a thematic analysis using the i‐PARIHS constructs (context, recipients, facilitation and innovation as described above) to develop a priori parent codes before beginning data analysis. As the team reviewed the transcripts, we identified additional a priori child codes based in the definitions and details of the i‐PARIHS constructs. For example, we created a child code for ‘state/federal regulations’ under external context construct. As coding progressed, we identified more child codes based on the i‐PARIHS framework.^33^, ^34^, ^35^ For example, a new child code was added for the internal context for ‘organisation culture’.

To conduct the coding, for three *foundational interviews* (see Table 1) an iterative consensus coding process involving the lead researcher and a research assistant was utilised to develop the initial codebook.^32^ After achieving consensus, the additional *foundational interviews* were then coded solely by the lead investigator. A similar consensus coding approach occurred for all *launch event data* sources and *initial evaluation interviews* utilising the initial codebook. Following the consensus process, transcripts of the *evaluation interviews* were divided between the lead investigator and a research assistant, with regular coding meetings to discuss difficult passages. A detailed logbook was used to track adaptations to the codebook. NVivo‐12 software was utilised.

After coding completion, the lead author developed preliminary themes and created a matrix with preliminary themes organised by data source and construct. Next, overarching themes for each construct were developed from the comprehensive analysis of all data points. The themes were member checked with the larger codesign planning group that had WWLE members.

## 3 RESULTS

### 3.1 Description of the study sample

The full sample across all stages of the EBCD implementation totalled 34 unique individuals (WWLE = 13 and FFNE‐p = 21) (Table 2).

**Table 2:** Sample demographics (*five participants' data missing).

Category	FFNE‐p* (*n* = 16)	WWLE (*n* = 13)
Age		
25–34	2 (13%)	6 (46%)
35–44	1 (6%)	5 (38%)
45–54	6 (38%)	2 (15%)
>55	7 (44%)	
Race		
White	16 (100%)	12 (92%)
Asian/Asian American		1 (8%)
Latina/Latino		
No	16 (100%)	13 (100%)
Number of years in recovery		
2–3		5 (38%)
3–5	1 (6%)	2 (15%)
>5	1 (6%)	5 (38%)
Not applicable/no answer	16 (88%)	1 (8%)
Highest education level		
H.S. degree		3 (23%)
Some college		7 (54%)
College degree	1 (6%)	2 (15%)
Graduate degree	15 (94%)	1 (8%)

Abbreviations: FFNE, Families Flourish Northeast; WWLE, women with lived experience.

### 3.2 Themes

Analysis revealed eight themes presented below by i‐PARIHS construct.

#### 3.2.1 Innovation

*Theme 1*: The core components of EBCD support the creation of a trusting environment that effectively engages all participants to collaboratively codesign.

Core aspects of the EBCD model are initially gathering and then sharing both service user and provider experiences at a launch event and then creating ongoing, small workgroups to tackle improvement initiatives. Grounding the codesign in shared experiences creates a trusting environment and enhances collaborative brainstorming. The launch event featured quotes from foundational interviews describing the experiences of both providers and WWLE with SUD treatment. Launch event participants felt hearing these quotes increased their empathy for women living through addiction and the treatment system.

I think what sticks in my mind most is just the quotes from the women. I think that was the most powerful part of that day, and that's what I remember the most, … and having women there that have lived experience reading the quotes. I think that really personalized things and made it powerful. (FFNE‐p #30)

The quotes provided a starting point for the group conversations. WWLE built on the quotes, freely sharing their own individual experiences, ranging from relapses to forced separation from their children. The collective sharing of experiences sparked group brainstorming, collaboratively building ideas for programme development. The brainstorming focused on a variety of issues from the hiring and selection process, the fine balance of freedom versus rules and structure, and creating a safe space for women to have ‘power over their decisions’.

Because my perspective of some of those quotes is different … how a clinician and a peer‐based support person view a comment are quite different. So, just being able to kind of talk that out and be like, ‘Well, what does this mean … How is that helpful? How is that not helpful?’ (FFNE‐p #2)

The positive impact of sharing experiences was carried throughout the ongoing workgroup collaborations.

I can't emphasize the importance of the interviews with the women with lived experience and the richness that brought to us being able to define policies … it was pivotal. Again, the interviews and the quotes and also from the providers, it was so rich with information and really helped drive what we did. (FFNE‐p #5)

These small workgroups, with an even mixture of WWLE and professionals, enhanced meaningful participation. The small group size of 4–8 participants with equal mixture of WWLE and professionals, created an opportunity for all individuals to share significant contributions.

And they would always say, ‘oh, I didn't think of it like that.’ And it's like … You wouldn't think of it like that because you've never had to live like that … So, if they had questions, it was always in a respectful way and they always would end up saying, oh yeah, I guess I understand. I just didn't see it that way. (WWLE #9)

More equitable participation increased the comfort and diminished the concerns of WWLE about openly sharing.

I think that having some people there with lived experience, having some people there that can relate to the working side of working with addiction. I think just having a mix, it was nice to see different views and looking outside of the box. (FFNE‐p #5)

The mixture of participants allowed for different perspectives to be voiced as applicable depending upon the topic and allowed for both the clinical expertise and WWLE's input to be heard.

I think when you look at a facility that's going to be a clinical facility, right? You do weigh a clinician's voice a little bit heavier. But, I also think at other points in the conversations, a peer‐based person or someone with lived experience voice always weighed heavier, right? I think it was …, ‘What are we talking about and who has more experience with this?’ So, I don't think it was throughout the whole thing one voice was dominating everything. I think there was an ebb and a flow. (FFNE‐p #2)

#### 3.2.2 Context

*Theme 2*: Complex content complicates the codesign preparation.

Residential SUD treatment is a highly regulated domain with strict requirements for accreditation and insurance reimbursement necessitating close consideration in some of the workgroups. Preparing for the workgroups demanded significant planning time and a learning curve for initial document preparation to ensure materials adequately explained the content and enhanced opportunities for meaningful participation and workgroup output.

Just trying to describe and help people understand how a codesign process could work overall and then break that down in ways that a whole bunch of people … could understand … looking through the regulations, policy, all different things. So, it was in digestible chunks. It wasn't just overwhelming. (FFNE‐p #17)

*Theme 3*: Even within a supportive organisational culture for codesign the uncertainties of the early start‐up phase of an organisation hinders codesign.

Overall the organisation was supportive of codesign with all professionals and board leaders expressing commitment to the concept of codesign in the individual interviews and this commitment appeared in meeting documentation. WWLE expressed less anxiety about participating because the professionals involved were known for working on other projects in the area and truly listening to WWLE.

I think it's if you're have people who are in a treatment relationship with each other in a work group, that's not necessarily going to give you all the honesty that you might want, on the other hand, it might also give some trust or sense of safety based on that relationship. (FFNE‐p #94)

For FFNE, professionals willingly volunteered significant time and energy but highlighted that other professional requirements diminished their ability to provide more focused contribution. Since participants were not employees, the organisation could not influence their availability.

The uncertainty about FFNE's start‐up timeline created shifting priorities for the key committees and ensuing areas of focus for the workgroups. Commitment to creating policies and processes through codesign was high, but incomplete details regarding the physical space, staffing, and a concrete timeline until opening, complicated the process. The focused content for the workgroups required significant preparation, making it difficult to respond quickly to the unfolding timeline.

I think the delay will give more time to build the right policy process … and procedures for the actual place that might have been kind of rushed now … Or the work group stuff might not have ever made it into a real policy and procedure development. So, I think that there's a better chance now of it really coalescing together. (FFNE‐p #17)

#### 3.2.3 Facilitation

*Theme 4*: Careful selection of skilled facilitators with pre‐existing relationships with participants supported WWLE engagement in the codesign process.

For the WWLE, having a pre‐existing relationship with the workgroup facilitator, especially as a peer recovery coach, eased participation concerns. These historical relationships also allowed facilitators to easily use names, ask informed follow‐up questions, and draw out different perspectives that enriched group discussions. These skilful, targeted requests for WWLE input created opportunities for contribution and built confidence.

They would ask the people with lived experience … our opinion before they would ask somebody that was quote unquote a professional in the field. So, they were always trying to see what we thought first and were really good about that. So, that built my confidence to really be able to speak my mind and know that they didn't think any less of me because of what I was saying. (WWLE #9)

*Theme 5*: A cohesive, multidisciplinary codesign planning group inclusive of WWLE improves the codesign process.

The codesign planning group met weekly at points during the implementation before the launch events. The mixture of personalities, passionate commitment to codesign, and quantity of time spent together cultivated a cohesive, productive group.

They're very good at making everybody feel equal. We are equal. They shouldn't even have to make you feel equal, but they don't give you the impression that they think they're better. They definitely know that there's a lot of stuff they don't know because they don't have the lived experience. (WWLE #47)

Having a committed group of clinical providers, staff, a communications specialist, and WWLE‐enriched content development for the workgroups. The WWLE provided strategic insights for specific language adaptations away from triggering language, such as renaming ‘blackout period’ as ‘self‐care period’.

I feel like we just worked so well together. And like I said, having [communication specialist] was a big help. When we needed to have visual things, she was really good at making it clean, very easy to understand, easy for everybody to work on separately and then come back together and go over what we had talked about. And we spent a good amount of time together. So, I think that the mutual respect was always there … And it was fun to do all of the stuff that we did together. (WWLE #9)

*Theme 6*: Utilising a remote platform impeded human connection and relationship building.

Feelings about remote participation were mixed, with almost all preferring in‐person. The initial intent was for a fully in‐person process, but the realities of the Covid‐19 pandemic forced remote implementation. Many participants appreciated the convenience of flexible scheduling and a shorter time commitment for workgroup participation, especially due to driving distances in a rural setting. A few participants from both cohorts felt less anxiety in a group virtual setting with new people or providers.

I'm a little bit more comfortable sharing my thoughts via remote, … but there's pros and cons. I'm not sure how to explain it … for me in person I've always been like a timid person, I guess. And being in person, I feel more vulnerable because people can see all of your cues, all of your nervousness and being remote, they can't. (FFNE‐p #19)

On the other hand, the remote platform diminished interpersonal contact and group connection.

I think definitely really feeling like you are creating a relationship with the Moms with Lived Experience. … at least you got to see people in person and really acknowledge them as a human being, as opposed to either a face on a screen. Or often if people had trouble with their video, you only heard their voice. (FFNE‐p #1)

The overwhelming majority of participants stated a preference for in‐person group activities for the ease of communication without Wi‐Fi struggles, the ability to read body language, and the opportunity for organic side conversations. Remote participation also limited the use of certain EBCD brainstorming tools meant to support ideation and group cohesion.

So being remote kind of makes it really difficult to read where someone's … coming from. Because you can sound so different, especially when you're sharing your opinion, you want to be heard so you're coming off strong. So, you can sound like you're disagreeing with somebody else when maybe you're not. (WWLE, #7)

#### 3.2.4 Recipients

*Theme 7*: WWLE felt respected, emotionally safe to share, and empowered by their codesign participation.

WWLE reported feeling comfortable sharing their personal experiences and perceived their input was respected. WWLE's input were evident even in the launch event exchanges, where they readily shared details about their lives living with addiction. Reflecting on the workgroups, WWLE perceived their contribution was equally respected based on the responses from other participants and the facilitators.

I was able to add different perspectives from others, even if they were in similar situations, all of our experiences are a little bit different. So, it was empowering, it was powerful and exciting to be able to contribute in a way. (WWLE, #21)

The positive feelings of WWLE around contribution contradicted many voices of concern about the power dynamic before the actual workgroups.

They're coming to the table, not expecting to have an equal voice, so ways that they can be pleasantly surprised and feel like they're being heard. It's really empowering … They're grateful to be asked and often hesitant to speak up. (FFNE‐p #16)

WWLE expressed pride with participating in codesign and the opportunity to draw from their own personal struggles with addiction to improve other women's treatment experiences.

Just that through all my suffering and all my bad choices, maybe it wasn't all for nothing, maybe being a part of something like this will help other people down the line and then all the years I wasted and all the mistakes I've made, maybe it wasn't all for nothing. (WWLE #9)

*Theme 8*: For the providers codesign was conceptually attractive, but some resisted the equitable collaboration integral to EBCD.

WWLE unanimously supported the EBCD approach and the majority of FFNE participants enthusiastically expressed support, grasping the value of EBCD's small, equally represented workgroups and a more collaborative partnership. A few central individuals did not embrace EBCD and the time consuming, small, equally represented workgroups. Others were enthusiastic, but still described the process as WWLE providing ‘feedback’, not grasping the goal of a fully collaborative partnership.

Everybody wants the same thing. Just some people perceive codesign differently than what it really is, I guess. And so, there was always just a disconnect. But, I think that the people that were involved in the work groups and the planning group really did help the clinical committee see that this was important and it needs to be continued. And at one point, [name] said like we do codesign because we have women with lived experience on the clinical committee … So, I do think that codesign is seen and will continue … (FFNE‐p #19)

The creation of the tobacco policy provides an example of the challenges faced by experts equitably integrating WWLE's perspective. Experts advocated for a tobacco‐free treatment facility, but WWLE data and input highlighted this approach presented a significant barrier to treatment engagement. Eventually, a collaborative solution was crafted, allowing very controlled tobacco use and commitment to codesigning tobacco cessation programming.

**Figure 2:**
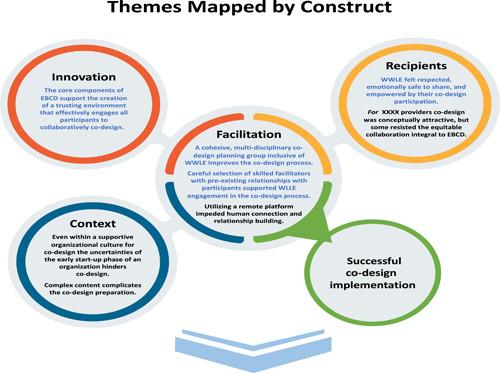
Themes mapped by construct.

**Figure 3:**
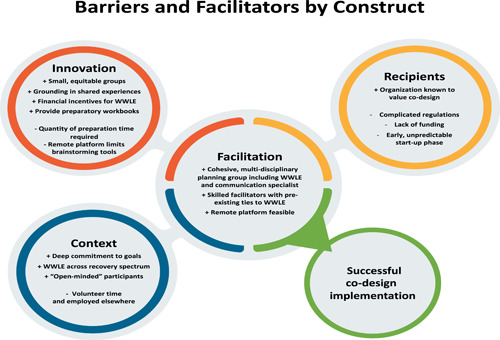
Barriers (−) and facilitator (+) mapped by construct.

The themes detailed above are presented in the following figures first by construct (Figure 2) and then broken down into specific barriers and facilitators (Figure 3). Figure 2 highlights which themes (those displayed in blue) from the different constructs supported a safe environment for meaningful participation: honest sharing and true collaborative problem solving.

## 4 DISCUSSION

These results highlight the feasibility of, and challenges to, creating a meaningful and extended codesign process that effectively engages WWLE. This research adds to EBCD literature and meets Green et al.'s^25^ definition of fidelity by including the gathering of experiences and ongoing collaborative workgroups with equal representation of the two cohorts.^25^ The identified themes reinforce the purposeful components within the EBCD process that enhance participation by creating a trusting environment. These components included gathering and sharing experiences and organising in ongoing small, equitably structured workgroups. New insights can inform successful codesign with a vulnerable population including the utilisation of a peer recovery coach as a codesign facilitator with strong pre‐existing ties with participants.

According to Harvey and Kitson,^30^ facilitation is the active influence on the other i‐PARIHS constructs that creates successful implementation. The themes detailed within the facilitation construct identify critical approaches for driving a successful implementation that were successful in overcoming some of the barriers.

In addition to the findings categorised under the facilitation construct, multiple cross construct factors were identified that enabled meaningful collaboration and a less unequal power dynamic in the workgroups. All cohorts involved anticipated that an unequal power dynamic might favour more input from the treatment providers, since participants expected WWLE would defer to providers or feel self‐conscious participating. However, the WWLE who participated in group work reported having an equal, respected voice which enabled true collaboration.^26^ WWLE attributed their comfort level with participation to the small, equally represented workgroups, skilled facilitation, pre‐existing relationships with the facilitator or other participants, and the reputation of FFNE professionals as ‘being people that really listen’. Additionally, having one of the internal workgroup facilitators be a peer recovery coach positively influenced both recruitment and comfort of the WWLE. Nuanced understanding of specific facilitation styles, such as being individually asked their opinion before providers, expands on existing research, and highlights the importance of skilled facilitation.^36^, ^37^ Other aspects important to WWLE reinforce previous findings regarding the value of small workgroup size, but our findings add insights into the importance of pre‐existing relationships and type of individual facilitator.^38^, ^39^ Replicating the benefits of a peer recovery coach facilitator with pre‐existing relationships could be a implementation barrier if no comparable staff exists. Consistent with previous EBCD literature, this research found individual benefits for the WWLE as they expressed empowerment from greater confidence and the opportunity to influence a project important to them.^40^

While the general concept of codesign had a high degree of fit in the organisation, the focus on equitable collaboration was a shift from existing practices of focus group style or tokenistic representation on a committee. Consistent with other codesign research, the shifting of this mindset is one of the most difficult barriers to overcome.^37^, ^41^ As explored by Dimopoulis‐Bick et al.,^42^ the challenges of some professionals recognising and breaking down engrained institutional and provider decision‐making ownership were evident in our findings. Once underway, EBCD met some resistance to truly collaborative work due to the reticence of a few key leaders at the organisation, which highlighted a barrier to the implementation of this innovative model.

The results show the feasibility of a codesign workgroup addressing complicated clinical workflow processes or specific policies affected by external regulations and guidelines. This influence has been explored in other research that illustrates the difficult, but necessary balance between research‐informed clinical expertise, regulations, and integrating individual's experiences.^43^ The planning and breakdown of complicated material allows nonexperts to have thoughtful input but requires the professionals and traditional experts to openly engage and reconsider current practices.^13^ These results should discourage using the excuse of complicated material as a reason to avoid a codesign approach.

The start‐up phase of organisation development and a shifting timeline for the residential opening presented barriers. Theoretically, a start‐up with strong ties to the target population presents an ideal contextual opportunity for codesigning from the inception. However, in this situation the uncertainties of start‐up became a challenge with uncertain timelines and lack of a known physical location limiting the EBCD adoption. Existing EBCD literature addresses improving or adding services within an established organisation.^36^, ^44^, ^45^, ^46^ This research adds to the contextual aspect of implementing EBCD within a newly formed organisation that is improving a service delivery system of care. This new context drove some of the decision to compress the original model and abide by the recommendations in Green et al.^25^ to maintain the core components of experience gathering and ongoing workgroups, along with the shift from video due to the vulnerable population as proven effective by Larkin et al.^37^ The successful implementation and positive experiences of both WWLE and FFNE professionals reinforce the feasibility of using EBCD in service creation and other adaptations.

## 5 STRENGTHS AND LIMITATIONS

Multiple data sources and intimate knowledge of local context supported rich data analysis. The strength of this research is the representation of WWLE at different stages of recovery, coupled with the integration of the provider perspective. WWLE were involved in the full scope of the research process, the codesign implementation, evaluation and presentation of findings. One of our coauthors (C. B.) is a WWLE who provided guidance on recruitment strategies, was a member of the codesign planning group and one of the work group facilitators. She also collaborated on ensuring the interview guide encompassed important questions with nonstigmatising language and on the presentation of the results crafting language and interpreting certain phrases or colloquialisms used by WWLE. Additionally, the insights from successfully working with WWLE on a remote platform can inform other projects with vulnerable populations. The primary limitation of this research is the small sample size of almost completely white women and a single location, which impacts generalisability, but was reflective of the local rural demographics.

A component that was both a strength and limitation were the lead researcher's dual role in the implementation: a strength given the insider perspective the investigator brought to the research and a limitation of the risk of personal bias in data collection and analysis. To mitigate this limitation, the research assistant conducted the evaluation interviews, the transcripts were deidentified, the lead investigator and the research assistant codeveloped the codebook and conducted the analysis, and the lead researcher wrote reflexive memos. Additionally, participants from both cohorts were given the opportunity to provide feedback on the findings.

## 6 CONCLUSION

This research demonstrates how the components of EBCD can create a collaborative codesign process between seemingly imbalanced groups of treatment providers and WWLE. The following core factors led to successful implementation of the codesign: participants' deep commitment to the project's values and goals, the effective codesign planning group, and excellent internal facilitators with pre‐existing comfort and relationships with the WWLE. These benefits allowed success even with the shift to a remote platform, a prolonged organisational start‐up phase, and funding challenges. Additionally, the findings demonstrated a positive influence on a WWLE's feelings of empowerment from participating in codesign.

Future research of adapted EBCD with this population should further explore the impact of peer recovery coaches as codesign facilitators, engaging WWLE earlier in the recovery phase throughout the ongoing workgroups and implementing an adapted EBCD in a system already providing services. More research is needed to evaluate longer term codesign outcomes such as the actual implementation of the organisational policies created in the codesign process and the impact on treatment engagement and retention. Overall, these findings should provide guidance to similar initiatives that aim to empower WWLE and collaboratively improve the current inequitable addiction treatment framework into a more patient‐centred model.

## CONFLICT OF INTEREST STATEMENT

The authors declare no conflict of interest.

## ETHICS STATEMENT

This research was approved by both Boston University Medical Campus and Dartmouth Hitchcock Medical Centre IRB as exempt Human Subjects research.

## Supporting information

Supporting information.

## Data Availability

Research data are not shared.
