# Supplementary material for: ‘It opened my eyes, my ears, and my heart’: Codesigning a substance use disorder treatment programme
Source: Health Expect. 2023 Nov 3;27(1):e13908. doi: 10.1111/hex.13908 (PMC10726284; doi:10.1111/hex.13908)
Supplement: Supplementary file 1 — Supporting information. [file HEX-27-e13908-s001.docx]

**Adapted EBCD supplemental material**

The full EBCD process specifies eight action stages (Donetto et al, 2014, Springham et al, 2015). The following table identifies the original 8 stages with the rationale for exclusion or adaptation with the FFNE implementation.

| **Original Full EBCD process** | **Rationale for adaptation with XXX** |
| --- | --- |
| **Stage 1:** Ethnographic observation of the existing clinical processes. | No existing service to observe and previous research showed acceptability of excluding this time consuming stage (Chisolm et al. 2018, Cooper et al, 2016). |
| **Stage 2:** Video recorded interviews eliciting direct personal experiences of service users and staff. Deeply understanding the user experience reveals how users interacted with the system. EBCD calls these interactions, “touchpoints,” defined as “polarizing moments when experiences are powerfully shaped. | The team did not have the funding and skill to create a compilation of video interviews.  Previous research that eliminated the video aspect had shown acceptable alternative approaches for collecting and presenting experiences (Green et al, 2020, Larkin et al, 2015). |
| **Stage 3:** An amalgamated video presentation is crafted to highlight themes or “touchpoints.” | Similar rationale as stage 2 |
| **Stages 4 and 5**: Theme group feedback events for the two cohorts start with the video presentation to build connection between participants and stimulate discussion of improvement opportunities. Facilitated discussion, aided by brainstorming tools, stimulates new ideas and builds group consensus on improvement priorities. | The co-design planning group collaboratively crafted the findings and materials for the launch event.  Given the constraints of Covid and the remote platform this stage was removed. |
| **Stage 6:** Launch events brings together both groups for another facilitated discussion, utilizing engagement tools and finalize improvement priorities. Facilitation and purposeful tool application aim to neutralize the power differential between service providers and service users ensuring users provide input. | Two launch events were conducted due to the remote platform it was anticipated that smaller groups with more equal representation would create a more collaborative environment (Mulvale et al, 2016). |
| **Stage 7:** Small ongoing co-design workgroups, ideally of equal representation from both service providers and service users. These ongoing workgroups typically meet over several months to collaboratively address the improvements. | This was fully implemented consistent with Green et al (2020) recommendations. |
| **Stage 8:** Celebratory event with all EBCD participants to reflect on accomplishments. | At completion of the first set of workgroups the constraints of the pandemic still required remote meetings for research purposes. This event was postponed until a later point and following more rounds of workgroups. |

**References**

Chisholm, L., Holttum, S., & Springham, N. (2018). Processes in an Experience-Based Co-Design Project With Family Carers in Community Mental Health. SAGE Open, 8(4). <https://doi.org/10.1177/2158244018809220>

Cooper, K., Gillmore, C., & Hogg, L. (2016). Experience-based co-design in an adult psychological therapies service. *Journal of mental health (Abingdon, England)*, *25*(1), 36–40. https://doi.org/10.3109/09638237.2015.1101423

Donetto, S., Pierri, P., Tsianakas, V., Robert, G. (2015) Experience-based co-design and healthcare improvement: realising participatory design in the public sector. *The Design Journal*, 18(2):227-248. DOI:[10.2752/175630615X14212498964312](http://dx.doi.org/10.2752/175630615X14212498964312)

Green, T., Bonner, A., Teleni, L., Bradford, N., Purtell, L., Douglas, C., Yates, P., MacAndrew, M., Dao, H. Y., & Chan, R. J. (2020). Use and reporting of experience-based codesign studies in the healthcare setting: A systematic review. *BMJ Quality & Safety*, *29*(1), 64–76. https://doi.org/10.1136/bmjqs-2019-009570

Larkin, M., Boden, Z. V. R., & Newton, E. (2015). On the Brink of Genuinely Collaborative Care: Experience-Based Co-Design in Mental Health. *Qualitative Health Research*, *25*(11), 1463–1476. https://doi.org/10.1177/1049732315576494

Mulvale, A., Miatello, A., Hackett, C., & Mulvale, G. (2016). Applying experience-based co-design with vulnerable populations: Lessons from a systematic review of methods to involve patients, families and service providers in child and youth mental health service improvement. *Patient Experience Journal*, *3*(1), 117–129. https://doi.org/10.35680/2372-0247.1104

Springham, N., & Robert, G. (2015). Experience based co-design reduces formal complaints on an acute mental health ward. *BMJ quality improvement reports*, *4*(1), u209153.w3970. https://doi.org/10.1136/bmjquality.u209153.w3970
